# Laparoscopic Posterior Cruroplasty and Anterior Gastropexy for Type IV Hiatal Hernia Repair in an Elderly Patient: A Case Report and Review of the Literature

**DOI:** 10.7759/cureus.46698

**Published:** 2023-10-08

**Authors:** Talal A Almutairi, Feras Alsannaa, Abdulaziz Altamran, Faisal Alnefaie

**Affiliations:** 1 General Surgery, Prince Sultan Military Medical City, Riyadh, SAU

**Keywords:** type iv hiatal hernia, laparoscopic posterior cruroplasty and anterior gastropexy, anterior gastropexy, posterior cruroplasty, hiatal hernia repair, paraesophageal hernias

## Abstract

A hiatal hernia describes a defect of the portion of the esophageal hiatus of the diaphragm, which leads to herniation of the abdominal contents into the chest cavity. Type IV paraesophageal hernias (PEH) have been associated with relatively large defects and are usually symptomatic. Surgical intervention is indicated in patients with symptoms or complicated paraesophageal hernias. The elderly age group represents a challenge in terms of management approach. Our purpose is to emphasize the safety and efficacy of early laparoscopic posterior cruroplasty and anterior gastropexy during PEH repair in the elderly age group.

A 90-year-old male without significant past medical or surgical history was admitted for a five-day history of left upper quadrant abdominal pain associated with multiple episodes of vomiting. The physical exam revealed left upper quadrant pain and rebound tenderness. Abdominal CT with IV contrast showed a large hiatal hernia containing the entire stomach and part of the duodenum with an abrupt transition zone at the duodenum. The patient underwent laparoscopic hiatal hernia repair, posterior cruroplasty, and anterior gastropexy. Postoperatively, the patient tolerated the procedure, and further follow-up in the clinic showed resolution of his symptoms without complications.

Prompt identification and proper management represent a crucial step in the management of PEH, especially in elderly comorbid patients. Laparoscopic anterior gastropexy is a safe and effective method for type III/IV hiatal hernias in elderly patients.

## Introduction

A hiatal hernia describes a defect of the portion of the esophageal hiatus of the diaphragm, which leads to herniation of the abdominal contents into the chest cavity. Four types of hiatal hernias have been described. It is estimated that greater than 95 percent of hiatus hernias are type I (sliding), with type II, III, and IV (paraesophageal) hernias accounting for approximately five percent [1]. Furthermore, type IV paraesophageal hernias (PEH) have been associated with a relatively large defect in the phrenoesophageal membrane with subsequent abnormal laxity in gastrosplenic and gastrocolic ligaments, which results in the stomach along with other abdominal organs being herniated into the chest cavity [2]. Patients with paraesophageal hernias can be asymptomatic or may present with ambiguous, intermittent symptoms, the most common of which are epigastric abdominal pain, postprandial fullness, nausea, and retching [1]. In terms of management, surgical intervention is indicated in patients with symptoms or with complicated hiatal hernia; the urgency of surgical intervention depends upon the type and time of presentation. However, emergency surgical repair is mandated in patients with acute presentation of gastric volvulus, uncontrolled bleeding, obstruction with or without strangulation, perforation of the herniated bowel, or acute respiratory symptoms [3]. Our purpose is to emphasize the safety and efficacy of early laparoscopic posterior cruroplasty and anterior gastropexy during paraesophageal hernias repair in elderly age group patients.

## Case presentation

**Figure 1:**
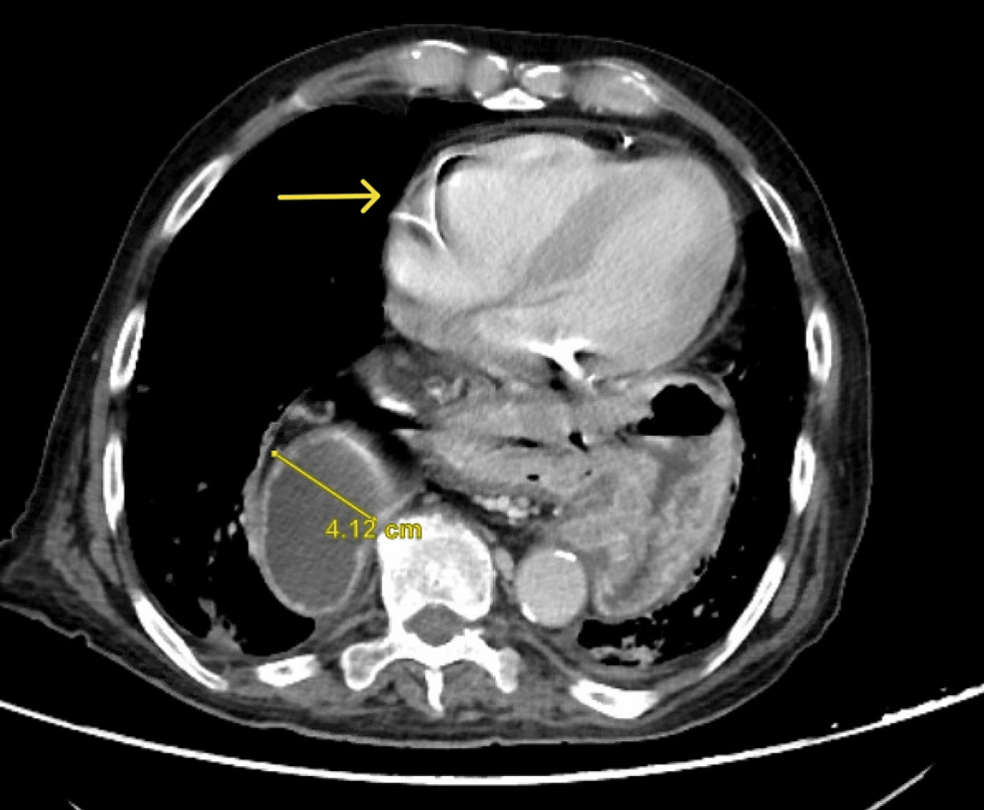
Abdominal CT with contrast (axial view) The image shows a large hiatal hernia containing the entire stomach and part of the duodenum (arrow) with an abrupt transition zone at the duodenum.

**Figure 2:**
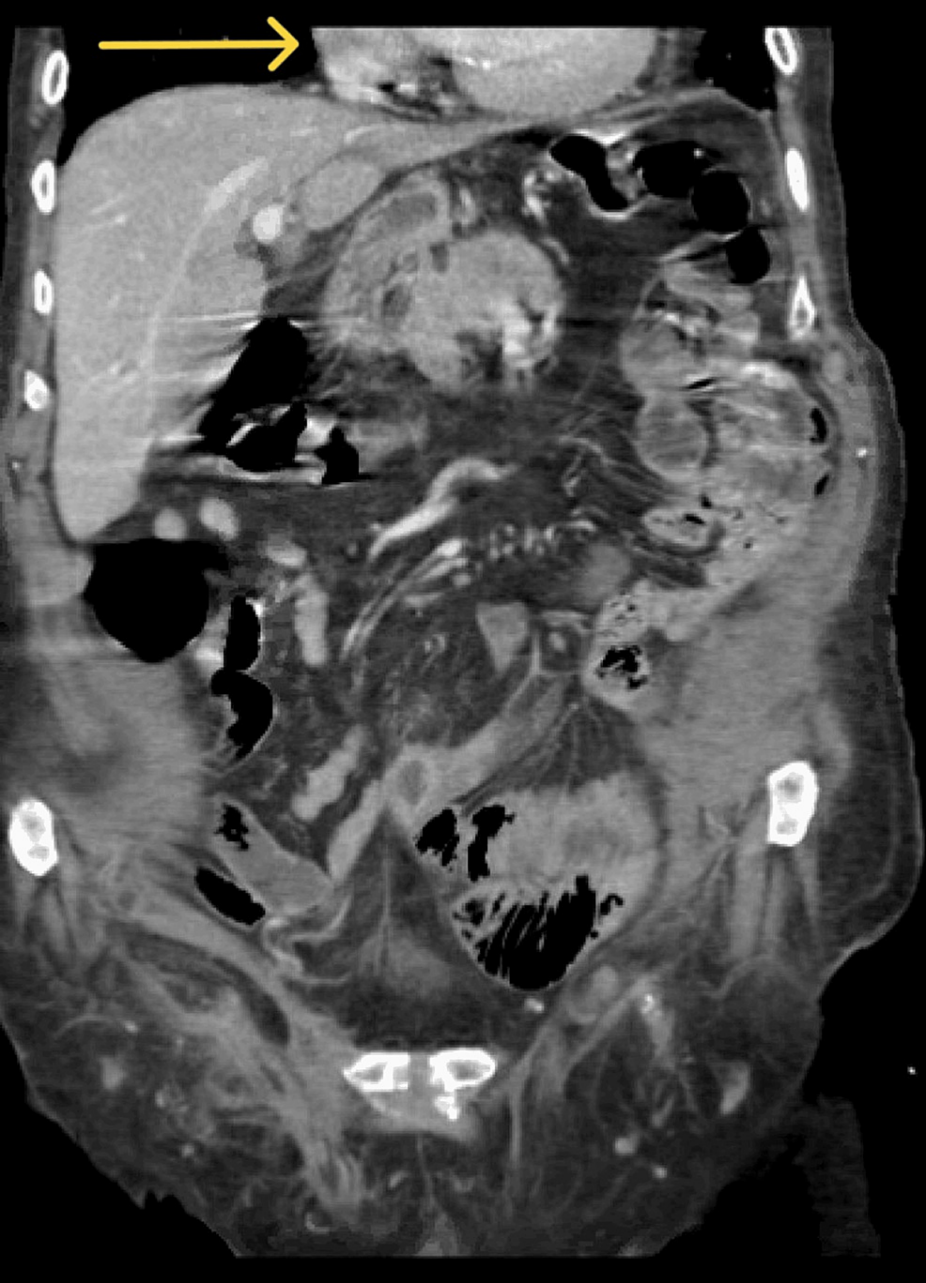
Abdominal CT with contrast (coronal view) The image shows another view of a large hiatal hernia containing the entire stomach and part of the duodenum (arrow) in the chest cavity.

**Figure 3:**
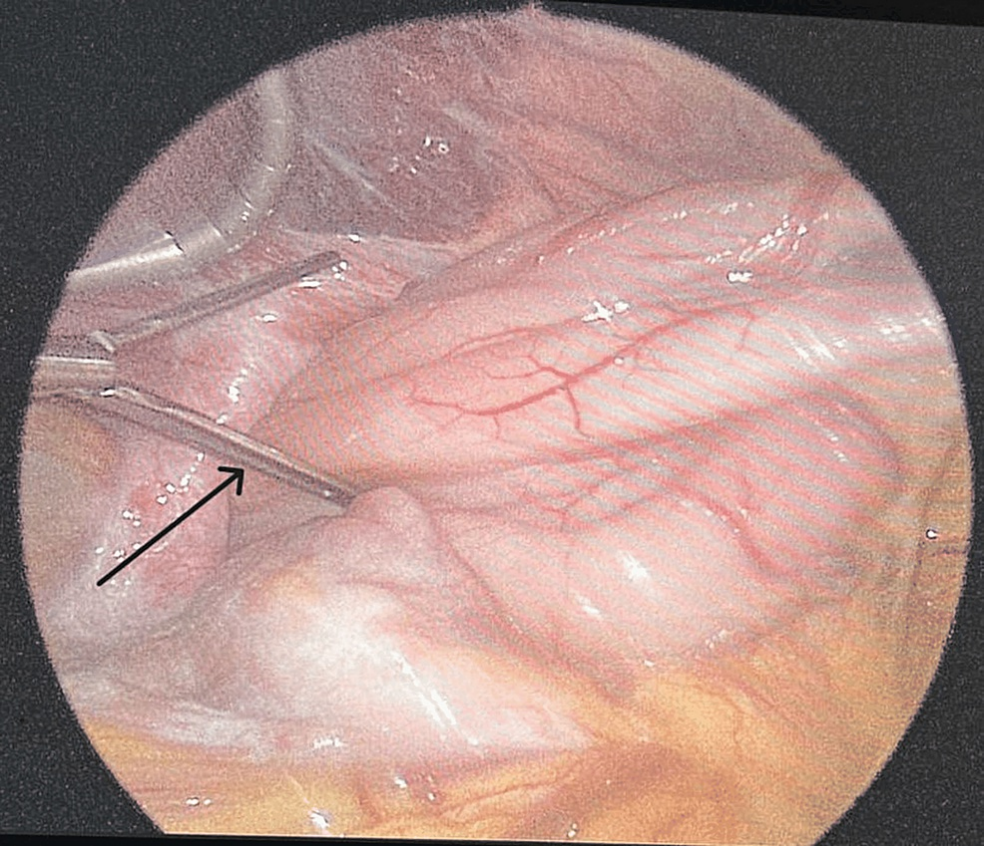
Laparoscopic view of the incarcerated hiatal hernia The image shows the herniated and twisted stomach and part of the small bowel through the defect (arrow).

**Figure 4:**
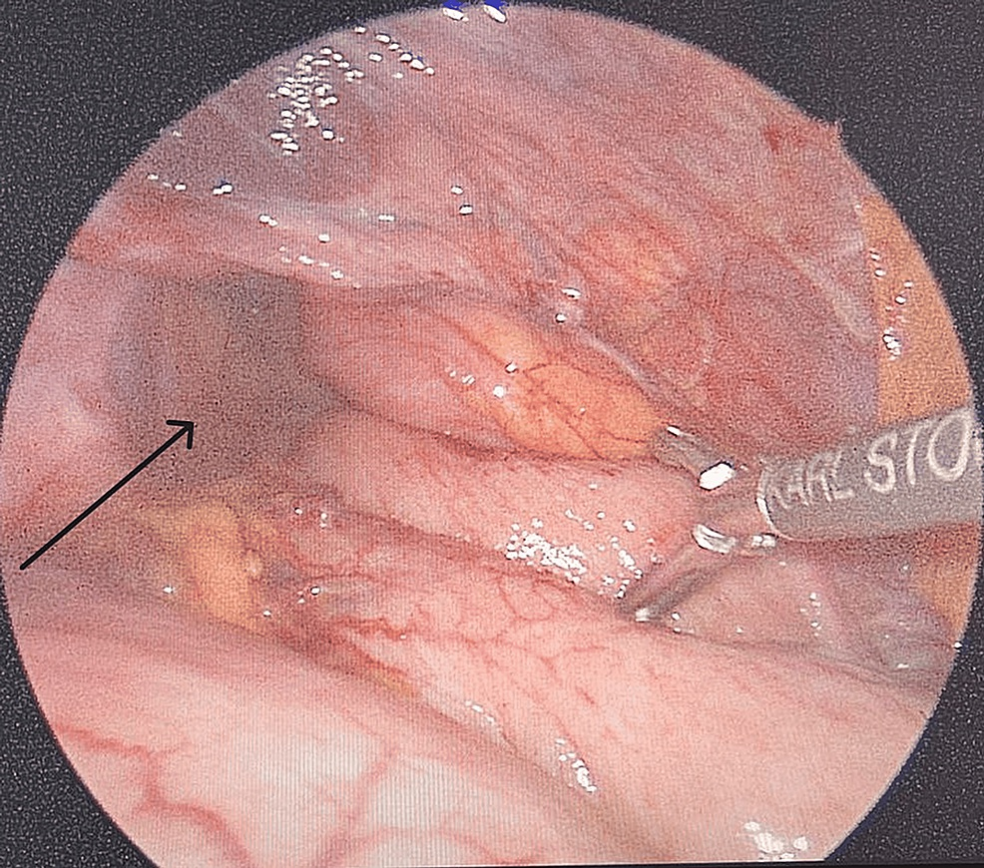
Laparoscopic view of the hiatal hernia defect The image clearly shows the large hiatal hernia defect prior to repair (arrow), again noting the twisted stomach with a part of the duodenum adherent to the hernial sac.

A 90-year-old male without significant past medical or surgical history was apparently healthy until presenting to the emergency department with a five-day history of left upper quadrant abdominal pain. This sudden increase in pain was associated with multiple episodes of vomiting. The patient denies a history of fever, rectal bleeding, or melena. Upon physical examination, the patient was in significant distress due to his pain. His vitals were within normal limits. His abdomen was soft but very tender on the left upper quadrant with associated rebound tenderness. His labs are unremarkable, with normal lactic acid levels. Abdominal CT with contrast was conducted and showed a large hiatal hernia containing the entire stomach and part of the duodenum with an abrupt transition zone at the duodenum (Figures 1, 2). The patient then underwent laparoscopic hiatal hernia repair, posterior cruroplasty, and anterior gastropexy. Intraoperative exploration showed an incarcerated hiatal hernia, which contains a twisted viable stomach with a part of the duodenum adherent to the sac (Figures 3, 4). The patient recovered without complications, returned to enteral nutrition, and his bowel function was restored. He was subsequently discharged on postoperative day six. He followed up in the clinic without exhibiting any signs or symptoms of recurrence.

## Discussion

Paraesophageal hernias account for approximately five percent of all hiatal hernias. Although obesity is a well-known risk factor for developing hiatal hernia, advanced age, on the other hand, has been found to be a predominant risk factor in different parts of the world [4]. Most patients with PEH are asymptomatic; however, when symptoms are present, patients usually complain of ambiguous symptoms such as epigastric abdominal pain, nausea or vomiting, and shortness of breath that is a result of obstruction to the lungs. Other complaints might include gastroesophageal reflux disease and anemia resulting from iron deficiency. [5]. Furthermore, this might explain the late presentation, especially in the elderly age group. Surgical repair is an effective method in the management of symptomatic or large PEH; however, operative mortality is estimated to be 17% in emergency surgery and 1.38% even for elective surgeries [6]. Therefore, proper selection of patients along with comprehensive perioperative assessment provides better outcomes. Laparoscopic hiatal hernia repair has proven itself to be an optimal surgical technique to achieve operative goals; however, large PEH type III/IV has been associated with an increased rate of asymptomatic recurrence as 40% of patients reoccur within one year after hernia repair alone [7,8]. Moreover, surgical management of paraesophageal hernias can be quite challenging, causing controversies between surgeons in terms of the proper surgical techniques and the decision to apply mesh [9]. Lower recurrence rates have been associated with the application of mesh during cruroplasty in comparison to choosing suture cruroplasty alone [9-11]. Although some studies favor the use of mesh over primary repair alone in terms of preventing recurrence, possible complications of such technique, including mesh infection, esophageal erosions, and stenosis, might balance the equation when deciding on the technique being used [5]. Furthermore, no significant difference was found in terms of quality of life in patients who underwent suture cruroplasty alone in comparison to those who underwent mesh herniorrhaphy, despite the use of radiological imaging to identify recurrence [12].

Our patient has advanced age, type IV hiatal hernia, and emergent presentation with twisted stomach, so we opted not to apply mesh in our case for a couple of reasons, one of which was that there are no guidelines or gold standard approach advocating the use of mesh in large paraesophageal hernias, and it is often left to the judgment and preference of the surgeons, especially in large paraesophageal hernias with emergent presentation [12]. Multiple studies have reported that laparoscopic anterior gastropexy is an effective and safe surgical option for hiatal hernia repair, especially in high-risk patients. Remarkable results and improvement of symptoms were found during a 48-month follow-up of eight patients who underwent laparoscopic paraesophageal hernia repair with anterior gastropexy in which they used interrupted suturing [4]. Obstructive and respiratory symptoms often improve after surgical intervention; 13 elderly patients with multiple comorbidities were found to have significant improvement of the above-mentioned symptoms during clinic follow-ups after they underwent laparoscopic paraesophageal hernia repair with anterior gastropexy [13].

## Conclusions

Prompt identification and proper management represent a crucial step in the management of paraesophageal hernias, especially in elderly comorbid patients. Significant improvement of obstructive and respiratory symptoms provides a better quality of life to such patients. Different surgical techniques can be used based on individuality and on a case-to-case basis. Laparoscopic anterior gastropexy and cruroplasty are generally considered safe and effective surgical options for type III/IV hiatal hernias in elderly patients.
